# Associations Among Resilience, Stress, Depression, and Internet Gaming Disorder in Young Adults

**DOI:** 10.3390/ijerph16173181

**Published:** 2019-08-31

**Authors:** Ju-Yu Yen, Huang-Chi Lin, Wei-Po Chou, Tai-Ling Liu, Chih-Hung Ko

**Affiliations:** 1 Department of Psychiatry, Faculty of Medicine, College of Medicine, Kaohsiung Medical University, Kaohsiung City 807, Taiwan; 2 Department of Psychiatry, Kaohsiung Municipal Ta-Tung Hospital, Kaohsiung Medical University, Kaohsiung City 812, Taiwan; 3 Department of Psychiatry, Kaohsiung Medical University Hospital, Kaohsiung Medical University, Kaohsiung City 807, Taiwan; 4 Department of Psychiatry, Kaohsiung Municipal Siaogang Hospital, Kaohsiung Medical University, Kaohsiung 801, Taiwan; 5 Substance and Behavior addiction Research Center, Kaohsiung Medical University, Kaohsiung City 807, Taiwan

**Keywords:** internet gaming disorder, stress, resilience, escape, depression

## Abstract

*Background and Aims*: Using gaming to escape emotional difficulty has been suggested to be a candidate mechanism contributing to Internet gaming disorder (IGD). This study evaluated the associations among resilience, perceived stress, depression, and IGD. *Methods*: A total of 87 participants in an IGD group and 87 participants in a control group were recruited into this study. IGD was diagnosed using the Diagnostic and Statistical Manual of Mental Disorders. Stress levels, resilience, and depression were measured by a self-reported questionnaire. *Results*: The IGD group had a lower resilience, higher perceived stress, and depression than the control group. Hierarchical regression analysis demonstrated that resilience was associated with IGD when perceived stress was controlled. After depression was controlled, resilience and perceived stress were not associated with IGD. Among the IGD group, those with low resilience had higher depression. Furthermore, discipline was the resilience characteristic associated with IGD. *Conclusions*: Low resilience was associated with a higher risk of IGD. IGD individuals with low resilience had higher depression. Depression was more associated with IGD than resilience. Depression assessments and stress coping interventions should be provided for individuals with IGD who exhibit low resilience or high stress.

## 1. Introduction

Internet gaming disorder (IGD) has been identified as a potential psychiatric disorder because of its negative effects on multiple domains of functioning [1,2,3]. The international prevalence ranges from 0.3% to 12%, and it is an increasing public health concern, especially in Asian countries. As well as cognitive and behavioral symptoms similar to those of substance-use disorder, IGD also reportedly has associations with psychiatric symptoms, including attention-deficit hyperactivity disorder, depression, anxiety, and psychosomatic symptoms [4,5,6,7]. High comorbidity of emotional symptoms suggests that individuals with IGD might use gaming to escape emotional difficulties [8,9]. 

Addictive behaviors are often initiated as a maladaptive mechanism for coping with stress [10]. Stress may enhance abstinent individuals’ memories of addictive behaviors as stress relievers then increase the risk of relapse to addictive behaviors after abstinence [11]. Tao et al. [12] were first to use the escape from stress through gaming as a criterion for Internet addiction. It was subsequently listed as a diagnostic criterion for IGD in the Diagnostic and Statistical Manual of Mental Disorders (DSM-5) [13]. “Negative escapism” describes gaming being negatively reinforced as a means of avoiding stress and was reported among 77.8% of internalizing patients [14]. Kim et al. [6] found escape from negative emotions to be associated with depression in IGD [9]. Furthermore, high levels of escapism were also reported to be associated with more IGD symptoms [15]. Moreover, the motives for escapism mediate the association between psychiatric distress and problematic online gaming [16]. Thus, negative escapism is associated with the symptoms of Internet addiction, psychological distress, and poor life satisfaction among massively multiplayer online role-playing gamers [17], and escapism under stress could play a major role in excessive gaming behavior.

Convincing evidence indicates that stress is a risk factor for addiction and triggers relapses [18]. The negative reinforcement model of addiction is defined as drug-taking or addictive behavior that alleviates a negative emotional state. According to some hypotheses, the negative emotional state that drives such behaviors as negative reinforcement is derived from dysregulation of brain stress systems involved in addiction processes [19]. Thus, investigating the perceived stress among adults with IGD could contribute to understanding its role in developing addictive online gaming behavior.

Not all individuals who face stress develop addictions. Therefore, resilience factors may protect these mentally healthy individuals [20]. For example, psychosocial resilience, such as positive emotions, optimism, humor, cognitive flexibility, and reappraisal could attenuate stress-induced psychopathology [21]. Thus, resilience was reported to be a buffering factor against Internet addiction [22]. Furthermore, resilience was reportedly lower among adolescents with IGD according to questionnaire assessments [15]. Its moderating role in the association between stress and IGD severity was demonstrated by an online questionnaire survey [23]. However, the difference in resilience was not evaluated among adults with IGD based on diagnostic interviewing. Further, resilience included a variety of personal characteristics, such as acceptance, problem-solving skills, capacity to recover, self-regulation, personal competence, and self-efficacy [23,24]. Which characteristics of resilience plays an important role involving development of IGD and should be intervened firstly has not been well-evaluated.

Depression is one of the most reported correlates of IGD [25]. Although the causal relationship had not been confirmed between depression and IGD, a longitudinal study suggested that depression could be an outcome of pathological gaming [26]. Prolong stressful events contribute to depression and resilience plays an important role in stress-related disorder and depression [27]. Resilience has been also reported to decrease the likelihood of stress-induced depression [21]. Both stress and depression were reported to be associated with IGD [23,25]. However, the associations among stress, depression, and resilience have not been well examined through interview studies among adults with IGD. Further, we might hypothesize that resilience could be associated with IGD and contribute to perceived stress and depression.

According to the aforementioned studies, resilience enables people to adapt successfully to stress or emotional difficulty and to avoid stress-related disorders [28]. Thus, we hypothesized that an individual with lower resilience could have higher perceived stress and depression. As IGD could be a maladjustment behavior to depression or stress, we hypothesized that individuals with low resilience were more likely to have IGD. Further, the higher perceived stress and depression could involve the association between low resilience and IGD. Moreover, among individuals of IGD, we hypothesized that those with lower resilience had higher perceived stress and depression. Thus, the aim of the study was to evaluate: (1) the difference in resilience, perceived stress, and depression between IGD and healthy controls; (2) the difference in depression and perceived stress between individuals with lower resilience and those with adequate resilience; (3) the confounding effect of perceived stress and depression in association between resilience and IGD; (4) the difference in depression and perceived stress between individuals with lower resilience and those with adequate resilience among the IGD group; and (5) the most associated resilience characteristics of IGD.

## 2. Methods

### 2.1. Participants

Our participants comprised individuals who had IGD at the time of the study (the IGD group) and individuals who had never had IGD (the control group) based on a design of case-control study. All participants were recruited by advertisement from September 2012 to October 2013. The criteria for the IGD group specified that participants be: (1) young adults aged 20 to 30 years and with more than 9 years of education; (2) individuals spending either ≥40 h per week or ≥4 h per day on weekdays and ≥8 h per day on weekends engaged in Internet gaming; and (3) individuals who maintained a pattern of Internet gaming for more than 2 years. Participants who met all of three criteria underwent an additional interview with a psychiatrist using the criteria of the DSM-5 [13] for IGD diagnosis. Those who had IGD at the time were classified into the IGD group.

For each participant in the IGD group, we matched a participant to be included in the control group by gender, education level, and age (within a range of 1 year). The recruitment criterion of participants in the control group was that their daily nonessential Internet use was less than 4 h. These participants were classified into the control group after a diagnostic interview with a psychiatrist.

All participants underwent the interview, which comprised two steps: (1) a diagnostic interview based on the Mini-International Neuropsychiatric Interview (MINI) to assess psychotic disorders, bipolar I disorder, and substance-use disorders; and (2) a history-taking interview to evaluate psychotropic medication use, mental retardation, severe physical disorder, and brain injury. Those who had psychotic disorders, bipolar I disorder, substance-use disorders, mental retardation, severe physical disorder, or brain injury or used psychotropic medication were excluded.

### 2.2. Measures

The diagnostic criteria of IGD were those of the DSM-5 [13]. The nine criteria comprised preoccupation, withdrawal, tolerance, unsuccessful attempts to control others, loss of interests other than gaming, continued excessive use despite psychosocial problems, deceit regarding online gaming, escape, and functional impairment [13]. We developed a semi-structured interview schedule to assess the DSM-5 criteria for IGD among our participants. Those who met five or more criteria for IGD were classified into the IGD group.

#### 2.2.1. Chinese Version of the MINI

We conducted a diagnostic interview based on the psychotic disorder, bipolar I disorder, and substance-use disorder modules in the Chinese version of the MINI [29] to detect those excluding psychiatric disorders.

#### 2.2.2. 14-Item Resilience Scale (R14)

The R14, which provides reliable internal consistency and external validity, was developed to evaluate the levels of resilience in the general population. Participants’ resilience was assessed using this scale [24], in which scores are calculated through summation of the response values for each item, enabling scores to range from 14 to 98. The internal consistency reliability (Cronbach’s alpha) of the total scale is 0.93 in the current study. Scores less than 65 indicate low resilience, scores between 65 and 81 indicate moderate resilience, and scores of more than 81 indicate high levels of resilience [24]. In this study, participants scoring more than 64 were classified into an adequate resilience group, and those scoring 64 or less were classified into a low resilience group.

#### 2.2.3. Perceived Stress Scale

The Perceived Stress Scale (PSS) was designed to measure the extent to which situations in one’s life are perceived as stressful. The PSS score was correlated with life-event scores, depression, and physical symptomatology [30]. It is suggested that the scale possesses adequate reliability for as outcome measure for experienced stress level. In this study, the 10-item PSS-10 was used to evaluate the level of stress experienced by participants and its internal consistency reliability (Cronbach’s alpha) was 0.86.

#### 2.2.4. Center for Epidemiological Studies’ Depression Scale

The 20-item Mandarin Chinese version [31] of the Center for Epidemiological Studies’ Depression Scale (CES-D) [32] is a self-administered evaluation of the frequency of depressive symptoms during the past week. This was used to evaluate depression. Its internal consistency reliability (Cronbach’s alpha) was 0.92 in the current study.

#### 2.2.5. Clinical Global Impression Scale for IGD

The Clinical Global Impression (CGI) scale [33] asks “Considering your total clinical experience with this particular population, how mentally ill is the patient at this time?” Possible responses were 1 for normal, not at all ill; 2 for borderline mentally ill; 3 for mildly ill; 4 for moderately ill; 5 for markedly ill; 6 for severely ill; and 7 for among the most ill patients. We modified the scale for IGD to 1 for normal, not at all ill; 2 for excessive online gaming without fulfilling the IGD criteria; 3 for fulfilling the IGD criteria with mild functional impairment; 4 for moderate functional impairment in health or one field such as academics, socializing, or profession; 5 for moderate functional impairment in multiple dimensions; 6 for severe impairment in one field; and 7 for severe impairment in multiple dimensions of daily life.

### 2.3. Statistical Analysis

We evaluated the associations of resilience, perceived stress, and depression with IGD using an independent *t*-test. The associations between gender, lower resilience, and IGD were evaluated through Chi-squared analysis. A hierarchical logistic regression model was used to evaluate associations of resilience, perceived stress, and depression with IGD. Finally, we evaluated the associations of resilience, perceived stress, and depression with CGI score among participants with IGD. A logistic regression evaluated the association between items of R14 and IGD with gender, age, and educational level controlled. A value of *p* < 0.05 was considered significant for all analyses, which were performed using the SPSS package (SPSS Inc., Chicago, IL, USA).

A total of 87 participants in the IGD group and 87 in the control group were enrolled in the study after informed consent was obtained. This study was approved by the Institutional Review Board of Kaohsiung Medical University Hospital (KMUH-IRB-990380).

## 3. Results

A total of 87 participants from the IGD group and 87 participants from the control group were enrolled in this study. No significant differences were exhibited in gender, age, or education level between the IGD and control groups, as revealed in Table 1. The IGD group scored lower on resilience, scored higher on perceived stress, and received a higher CGI score than the control group. When participants were further classified into the adequate and low resilience groups, participants with low resilience were more likely to be diagnosed with IGD. Furthermore, participants with low resilience exhibited higher perceived stress and depression, as shown in Table 2.

### 3.1. Associations Among Resilience, Perceived Stress, Depression, and IGD

The hierarchical regression analysis in Table 3 demonstrates that low resilience was positively associated with IGD in Model 1 with gender, age, and educational level controlled. Participants with low resilience had a higher odds ratio (OR, 3.46; 95% confidence interval (CI) = 1.72–6.97) for IGD.

Perceived stress was significantly positively associated with IGD in Model 2. As the Wald **χ**^2^ of perceived stress was higher than that of resilience, perceived stress was more associated with IGD than resilience was (Model 2 in Table 3). This result suggests perceived stress was another important factor to IGD. Further, the resilience was significantly associated with IGD with perceived stress controlled for. It suggested that low resilience had an independent association with IGD from perceived stress.

Depression was significantly positively associated with IGD in Model 3 (OR = 1.1, 95% CI = 1.04–1.17). Resilience and perceived stress exhibited no significant associations with IGD when depression was controlled for. This result demonstrated the mediating effect of depression in the associations of resilience and perceived stress with IGD. It also demonstrated that among resilience, stress, and depression, depression was the factor most associated with IGD.

### 3.2. The Difference in Perceived Stress and Depression between Individuals with Low Resilience and Adequate Resilience Among IGD Group

The *t*-test in Table 2 demonstrates that individuals with low resilience had higher depression among the IGD group (*t* = 0.023; *p* = 0.03). They also had a trend to have higher percieved stress (*t* = 1.85; *p* = 0.07); however, this did not reach significance.

### 3.3. Association of Resilience Characteristics with IGD

We firstly evaluated the difference in each item of R14 between IGD and the control group. It demonstrated that the IGD group had a lower score in ability to copy (*t* = 2.15; *p* = 0.03), acceptance (*t* = 2.63; *p* = 0.01), drive (*t* = 2.81; *p* = 0.01), discipline (*t* = 6.94; *p* < 0.001), interest/engagement (*t* = 2.88; *p* = 0.00), self-efficacy (*t* = 2.29; *p* = 0.02), dependable (*t* = 2.59; *p* = 0.01), meaning (*t* = 3.57; *p* < 0.001), and resourcefulness (*t* = 2.55; *p* = 0.01). Then, we regressed IGD on these significant items in R14 with gender, age, and educational level controlled. The logistic regression in Table 4 demonstrates that “I am self-disciplined” was the only resilience characteristic associated with IGD.

## 4. Discussion

### 4.1. Perceived Stress of Individuals with IGD

This study demonstrated that participants with IGD experienced higher perceived stress. This result corresponds with those of other studies [14,23]. Stress is conceptualized as a risk factor contributing to progressive long-term changes in the brain and a subsequent drug-prone state characterized by craving and increased risk of relapse [34]. Accordingly, our results might support the possible role of perceived stress in developing IGD addiction. IGD may also have negative consequences. Ko et al. [35] reported that 46.5%, 25.4%,19.7%, and 4.2% of participants with IGD experienced impaired social interaction, professional failures, impaired family relationships, and failures on examinations related to career opportunities, respectively. Such negative consequences may stress adults with IGD and explain the increased perceived stress levels in our study. Further prospective studies to investigate the addiction process may illuminate the causal relationship between stress and IGD.

### 4.2. Associations among Resilience, Perceived Stress, Depression, and IGD

As in the studies of Wu et al. [36] and Canale et al. [23], the interview in the present study demonstrated an association between resilience and IGD. Furthermore, we demonstrated that those with low resilience have a 3.46 times odds ratio of being diagnosed with IGD. This suggests that low resilience plays a risk role in online gaming addiction. In the R14, resilience is represented as a stable personal resource and a positive personality trait, such as discipline, that can contribute to personal competence, self-acceptance, and life satisfaction [37]. These characteristics may facilitate stress coping and attenuate stress-induced depression [21]. The lower perceived stress and depression among the adequate resilience group demonstrated in our results might support such a claim. Therefore, the characteristics of resilience can be promoted to prevent IGD and to attenuate perceived stress and depression among the general population.

Two studies have evaluated the buffering effect of resilience on the association between stress and IGD. Wu et al. [36] did not observe significant protective effects of resilience against the effects of stress on IGD. However, Canale et al. [23] identified a moderating role of resilience and demonstrated that higher perceived stress and lower resilience were associated with increased gaming time. The difference in stress measurement methods might have contributed to the inconsistent results. However, the assessment of IGD was performed using questionnaires in the two aforementioned studies. Our study, which used similar measurements to Canale’s [23], demonstrated that individuals with IGD had lower resilience and higher perceived stress. Our regression analysis also demonstrated that perceived stress had a higher association with IGD than resilience. Although resilience plays a role in the development of IGD, perceived stress may be another influential factor and should be a target of intervention to prevent IGD.

Individuals with IGD could experience the psychosocial distress results from excessive gaming [35]. However, individuals with IGD usually cope with stress through gaming [15], exhibiting escapist gaming. This might aggravate, not resolve, their psychosocial problems. In the current study, the within-group analysis demonstrated IGD individuals with low reliance had higher, but not significantly, perceived stress than those with adequate resilience. This suggests a limited buffering effect of resilience on stress among individual with IGD relative to that in the general population. This might suggest further intervention aside of promoting resilience should be provided to copy the stress associated with IGD. Therefore, alternative stress coping strategies, such as problem-focus coping, exercise, or mindfulness [38] should be provided to replace escapist gaming for IGD individuals under stress.

Depression is one of the most reported factors associated with IGD [39,40]. With depression controlled, perceived stress and resilience were not significantly associated with IGD. This suggests that depression is the most proximal factor contributing to IGD. According to Baron and Kenny [41], depression may play a mediating role in the association between resilience, perceived stress and IGD. Depression could be the outcome of IGD [26], as IGD leads to psychosocial stress. Without adequate resilience to buffer dysphoric mood and psychosocial stress, individuals of IGD can use gaming as an escape from depression. However, excessive gaming without limitation might lead to further psychosocial problems and result in a vicious cycle. Further, these results also suggest that depression as a mood status has a stronger association with IGD than perceived stress and resilience. This study demonstrated that individuals with IGD had lower resilience and higher depression. Thus, depression could also be the outcome of low resilience under high stress which then contributes to IGD. The causal relationship or bi-direction interaction among resilience, stress, depression, and IGD cannot be confirmed in this cross-sectional study. Nevertheless, comorbid depression should be well assessed and effective intervention—such as antidepressants, cognitive behavior therapy [42], exercise, or promoting resilience—for depression should be provided for individuals with IGD, particularly those with lower resilience or higher perceived stress. Although the depression could be the outcome of IGD [26], gaming could be used to temporarily escape from dysphoric moods for those with limited alternative coping strategies. Thus, depression should be well intervened before aggressive intervention for gaming behavior of individuals with IGD.

### 4.3. Resilience Characteristic Most Associated with IGD

Discipline was the only resilience characteristic associated with IGD when other items were controlled. Individuals with IGD had lower discipline than controls did. Self-regulation was reported to mediate the association between impulsivity and pathological video gaming among youth [43]. Individuals with lower discipline might have had difficulty in controlling their excessive gaming, leading to a risk of IGD. Conversely, a loss of control in gaming with negative consequences might represent a lifestyle with lower discipline. The causal relationship between discipline and IGD cannot be confirmed with a cross-sectional study. However, our study suggests it is important to promote discipline to prevent risk of IGD among the general population and to prevent the deterioration of daily life functioning among individuals with IGD. Future prospective studies regarding the predictive ability of discipline for IGD or the treatment effect of promoting discipline in IGD may clarify this causal relationship.

## 5. Limitations

This study had limitations that should be considered when interpreting the findings. First, IGD was evaluated only through a diagnostic interview with participants. Additional information from family members or partners could have increased the validity of the diagnosis. Second, the cross-sectional research design could not confirm causal relationships between resilience, perceived stress or escapist online gaming behaviors and IGD. Lastly, we excluded those take psychotropic medication from this study to prevent its effect on depression and perceived stress. However, it could limit the generation of this study’s results to populations with psychotropic medication.

## 6. Conclusions

Individuals with IGD had lower resilience, higher perceived stress, and higher depression. Participants with low resilience exhibited 3.46 times odds ratio for IGD compared with those with adequate resilience. Further, depression was a more proximal factor to IGD than resilience and mediated the association between resilience and IGD. Interventions to promote coping strategies and treat depression should therefore be provided for individuals with IGD and concurrent high stress or depression. Finally, discipline is the resilience characteristic most associated with IGD. Its implications for IGD interventions deserve further study.

## Figures and Tables

**Table 1:** Associations of age, educational level, gender, perceived stress, depression, Clinical Global Impression score, and resilience with Internet gaming disorder (IGD).

Variables	IGD Diagnosis		χ^2^ Test
	Yes (*N* = 87) *N*(%)	No (*N* = 87) *N*(%)	
Gender			
Female (*N* = 34)	17(50.0)	17(50.0)	0.000
Male (*N* = 140)	70(50.0)	70(50.0)	
Resilience			
Lower (*N* = 53)	37(69.8)	16(30.2)	11.97 **
Adequate (*N* = 121)	50(41.3)	71(58.7)	
	**Mean ± SD**	**Mean ± SD**	***t*-Test**
Age	23.29 ± 2.34	23.38 ± 2.40	0.26
Educational level	15.93 ± 1.15	16.14 ± 1.22	1.15
Perceived stress	21.71 ± 5.54	17.76 ± 5.62	−4.67 ***
Resilience	66.57 ± 12.72	73.06 ± 12.66	3.37 **
Depression	20.44 ± 10.00	12.01 ± 7.90	−6.16 ***
CGI score	4.56 ± 0.94	1.09 ± 0.29	−33.02 ***

**: <0.01; ***: <0.001. Resilience: score on 14-Item Resilience Scale; participants scoring 64 or less were classified into a low resilience group. Perceived stress: score on Perceived Stress Scale-10. Depression: score on Center for Epidemiological Studies’ Depression Scale. CGI: Clinical Global Impressions scale.

**Table 2:** The difference in perceived stress and depression between individuals with low resilience and those with adequate resilience.

Variables	Resilience		*t*-Test
	Low Mean ± SD	Adequate Mean ± SD	
Among all subjects	(*N* = 53)	(*N* = 121)	
Perceived stress	22.38 ± 5.87	18.58 ± 5.56	3.99 ***
Depression	21.77 ± 9.19	13.79 ± 9.28	5.24 ***
Among IGD group	(*N* = 37)	(*N* = 50)	
Perceived stress	22.97 ± 5.85	20.78 ± 5.16	1.85 ^a^
Depression	23.16 ± 9.30	18.42 ± 10.12	2.23 *

*: <0.05; ***: <0.001. Resilience: score on 14-Item Resilience Scale; participants scoring 64 or less were classified into a low resilience group. Perceived stress: score on Perceived Stress Scale-10. Depression: score on Center for Epidemiological Studies’ Depression Scale. ^a^: *p* = 0.07.

**Table 3:** Hierarchical logistic regression model of Internet gaming disorder for resilience, perceived stress, and depression controlling for gender, age, and education level.

Variables	Wald	Exp(β)	95% CI
Model 1			
Age (year)	0.39	1.05	0.91–1.21
Education level (year)	2.09	0.81	0.60–1.08
Gender	0.00	0.98	0.45–2.14
Resilience (Low)	12.08 **	3.46	1.72–6.97
Model 2			
Age (year)	0.40	1.05	0.90–1.22
Education level (year)	1.02	0.85	0.63–1.16
Gender	0.05	0.92	0.40–2.08
Resilience (Low)	6.08 *	2.53	1.21–5.30
Perceived stress	11.92 **	1.12	1.05–1.19
Model 3			
Age (year)	0.52	1.05	0.91–1.23
Education level (year)	1.51	0.82	0.60–1.13
Gender	0.00	1.01	0.43–2.35
Resilience	2.70	1.92	0.88–4.16
Perceived stress	0.03	1.00	0.91–1.09
Depression	9.83 **	1.10	1.04–1.17

*: <0.05; **: <0.01. Resilience: score on 14-Item Resilience Scale; participants scoring 64 or less were classified into a low resilience group. Perceived stress: score on Perceived Stress Scale-10. Depression: score on Center for Epidemiological Studies’ Depression Scale.

**Table 4:** Logistic regression model of Internet gaming disorder for items of 14-Item Resilience Scale with controlling for gender, age, and education level.

Variables	Wald	Exp(β)	95% CI
Model 1			
Age (year)	0.00	0.97	0.90–1.25
Education level (year)	0.45	1.06	0.56–1.11
Gender	1.85	0.79	0.40–2.33
Ability to cope	0.05	1.05	0.69–1.59
Acceptance	0.13	0.93	0.63–1.39
Drive	0.36	1.12	0.79–1.60
Discipline	24.44 ***	0.41	0.29–0.58
Interest/engagement	0.30	1.13	0.74–1.72
Self-efficacy	0.86	1.22	0.80–1.84
Dependable	0.02	1.03	0.67–1.58
Meaning	2.46	0.73	0.49–1.08
Resourcefulness	0.00	1.00	0.60–1.65

***: <0.001. Items of 14-Item Resilience Scale: the items were significantly associated with IGD in *t*-test evaluation.
